# Neuron populations across layer 2-6 in the mouse visual cortex exhibit different coding abilities in the awake mice

**DOI:** 10.3389/fncel.2023.1238777

**Published:** 2023-09-25

**Authors:** Chui Kong, Yangzhen Wang, Guihua Xiao

**Affiliations:** ^1^School of Information Science and Technology, Fudan University, Shanghai, China; ^2^Department of Communication Science and Engineering, Fudan University, Shanghai, China; ^3^Department of Automation, Tsinghua University, Beijing, China; ^4^BNRist, Tsinghua University, Beijing, China

**Keywords:** neuron, layer-specified, visual cortex, stimulus decoding, layers diversity, sensory input, cortical column, two-photon imaging

## Abstract

**Introduction:**

The visual cortex is a key region in the mouse brain, responsible for processing visual information. Comprised of six distinct layers, each with unique neuronal types and connections, the visual cortex exhibits diverse decoding properties across its layers. This study aimed to investigate the relationship between visual stimulus decoding properties and the cortical layers of the visual cortex while considering how this relationship varies across different decoders and brain regions.

**Methods:**

This study reached the above conclusions by analyzing two publicly available datasets obtained through two-photon microscopy of visual cortex neuronal responses. Various types of decoders were tested for visual cortex decoding.

**Results:**

Our findings indicate that the decoding accuracy of neuronal populations with consistent sizes varies among visual cortical layers for visual stimuli such as drift gratings and natural images. In particular, layer 4 neurons in VISp exhibited significantly higher decoding accuracy for visual stimulus identity compared to other layers. However, in VISm, the decoding accuracy of neuronal populations with the same size in layer 2/3 was higher than that in layer 4, despite the overall accuracy being lower than that in VISp and VISl. Furthermore, SVM surpassed other decoders in terms of accuracy, with the variation in decoding performance across layers being consistent among decoders. Additionally, we found that the difference in decoding accuracy across different imaging depths was not associated with the mean orientation selectivity index (OSI) and the mean direction selectivity index (DSI) neurons, but showed a significant positive correlation with the mean reliability and mean signal-to-noise ratio (SNR) of each layer's neuron population.

**Discussion:**

These findings lend new insights into the decoding properties of the visual cortex, highlighting the role of different cortical layers and decoders in determining decoding accuracy. The correlations identified between decoding accuracy and factors such as reliability and SNR pave the way for more nuanced understandings of visual cortex functioning.

## 1. Introduction

The mammalian visual cortex plays an essential role in visual information processing. It consists of six distinct layers, each possessing specialized functions that contribute variably to visual perception. In the visual cortex, neuronal populations exhibit variations in their tuning to visual stimuli at different depths. Hubel and Wiesel (1974) notably described this phenomenon through their identification of “orientation-selective columns”. These columns play a crucial role in preventing abnormal sensitivities to various line orientations within the visual field. In terms of the information transmission across different cortical layers, according to Gilbert and Wiesel's circuit posits (Gilbert, 1983), thalamic input reaches layer 4 (L4), and excitatory cells in L4 relay signals to the layer 2/3 (L2/3), which further project to layer 5 (L5) and then to layer 6 (L6). This loop is completed by a projection from L6 back to the input L4. The circuit has been extensively studied (Douglas and Martin, 2004; Olsen et al., 2012; Harris and Mrsic-Flogel, 2013; Quiquempoix et al., 2018; Marshel et al., 2019), forming the foundation for studies on the organization of the visual cortex and the collaboration and information transmission of cortical layers in processing visual information.

Mice are among the most widely used model organisms in mammalian visual cortex research. Transgenic cre-lines in mice facilitate the expression of genetically encoded fluorescent calcium sensors (Luo et al., 2008), thereby allowing the expression of fluorescent proteins and fluorescent-labeled biomolecules in live mammalian neurons (Giepmans et al., 2006). This ever-evolving technique permits researchers to image neural populations across different layers by expressing fluorescent proteins in the mouse visual cortex neurons, using either transgenic or viral approaches. Studying layer-specific performance requires three-dimensional imaging. Light field microscopy (LFM) offers a snapshot-based approach to three-dimensional imaging with low phototoxicity on single-photon microscopy. And the development of virtual-scanning LFM (VsLFM) has pushed the resolution to the diffraction limit of a snapshot by introducing periodic optical beam scanning to increase spatial sampling density (Wu et al., 2022; Zhang et al., 2022; Lu et al., 2023). In comparison to single-photon microscopy, two-photon microscopy (Helmchen and Denk, 2005) provides several advantages for observing neural activity, such as enhanced tissue penetration, reduced background noise, and improved resolution. Furthermore, recent advancements in two-photon microscopy have mitigated its typical limitations of a smaller field of view and high phototoxicity (Zhao et al., 2023). In this study, we examined two datasets: one sourced from the Allen Brain Observatory Visual Coding (Allen Institute MindScope Program, 2016; de Vries et al., 2020) and the other from Stringer's publication (Stringer et al., 2019a). Both datasets were acquired using two-photon microscopy.

Drift grating orientation classification and natural image classification are prevalent visual tasks employed to evaluate the stimulus tuning of neurons within the primary visual cortex. Neurons in the primary visual cortex process linear features such as orientations and temporal frequencies. This selectivity emerges from a blend of afferent input from the lateral geniculate nucleus (LGN) and intracortical inhibitory inputs (Xing et al., 2004). A plethora of studies has delved into the tuning properties of individual neurons in response to diverse stimuli, further investigating the decoding attributes of neuronal assemblies in the visual cortex. For example, researchers have classified tens of thousands of neurons based on their joint reliability to multiple stimuli using data from the Allen Brain Observatory, subsequently validating this functional classification via visual responses (de Vries et al., 2020).

Marshel et al. (2019) found thatthe activity of neural ensembles in the visual cortex typically propagates from L2/3 to L5rather than vice versa, and activating L2/3 necessitates stimulating a larger numberof cells compared to L5. Stringer et al. (2019a) demonstrated that large neuronal ensemblesrespond to high-dimensional natural image stimuli, resulting in high-dimensionalcollective activity with a power-law distribution of information across dimensions. Additionally, Stringer et al. (2019b) identified that neurons in the primary visual cortex concurrently encode visual-related information and motion-related activity associated with facial movements, implying the early amalgamation of sensory input and motion behavior within the primary sensory cortex. Stringer's research group also leveraged activity recordings from up to 50,000 neurons, gauged stimulus discrimination thresholds, and deduced that neural thresholds were almost 100 times more refined than behavioral discrimination thresholds in mice, signifying that perceptual discrimination in these animals is constrained more by non-sensory networks than by sensory representation neural noise (Stringer et al., 2021). Extensive research has been dedicated to understanding the decoding properties in neuronal populations. Concerning the study of various regions of the visual cortex, Marshel et al. (2011) analyzed the functional specialization of the mouse visual cortex's L2/3 and found that Higher visual areas are functionally distinct, with disparate groups of areas possibly specializing in computations related to motion and pattern processing. For decoder enhancements, Schneider et al. (2023) developed a decoding method that combines behavioral and neural data using either a supervised or self-supervised approach, revealing complex kinematic features. Many of the aforementioned studies have scrutinized the functional specialization of neuronal populations across various regions of the mouse visual cortex. However, there is a lack of research on the stimulus-specified, decoder-specified, and layer-specified properties across different cortical layers in decoding stimulus identity from neuronal populations of the same size. Consequently, probing decoding discrepancies via diverse stimulus categories, population dimensions, and varied decoders might unveil the intrinsic reasons for the disparate efficacy of neuronal groups in visual stimulus assignments.

The objective of this research was to discern potential variances in decoding visual stimuli based on the responses of neuronal assemblies across cortical layers within the mouse visual cortex and to elucidate the determinants behind these disparities. We incorporated a range of commonly used multi-class decoders and utilized the decoders used in the aforementioned studies in our research to analyze the decoding differences across layers and found diverse decoding differences among neuronal populations in L2/3 to L6. As the information is relayed to connected layers and secondary visual areas from the thalamus, associated and context-dependent information is added, we hypothesize that holding population size constant, the decoding precision of the neuronal population in L4 would be superior. We provide evidence confirming our hypothesis that differences exist in the decoding accuracy of neural populations across cortical layers.

## 2. Materials and methods

### 2.1. Visual stimulation two-photon dataset

In this study, we primarily utilized Allen Brain Observatory (Allen Institute MindScope Program, 2016) dataset to analyze the inter-layer differences in the mouse visual cortex in response to two visual stimuli and explore the factors contributing to these differences. We investigated the influence of decoder types and cell population size on decoding accuracy across different layers. Additionally, we validated the influencing factors behind the decoding differences across imaging depths using Stringer dataset (Stringer et al., 2019a).

If not specifically mentioned, all other data was included without quality control. When limiting the number of neurons, random multiple repetitions were used for selection, and the results were averaged. We have created a table to display the basic information of the two datasets (Supplementary Table 1). The table includes details on the GCaMP6 variants, cre-lines, number of experiments, average number of neurons, target cell of each cre-line, and their distribution across different cortical layers for both datasets.

#### 2.1.1. Allen Brain Observatory dataset

The Allen Brain Observatory dataset offers population imaging of neural activity across various brain regions and imaging depths, utilizing two-photon microscopy at a 30 Hz time resolution. This dataset comprises experimental results from the primary (VISp), lateral (VISl), anterolateral (VISal), anteromedial (VISam), posteromedial (VISpm), and rostrolateral (VISrl) visual areas from mice with different cell lines that enable the expression of GCaMP6f in different layers of the mouse visual cortex, thereby facilitating the study of decoding characteristics and performance across cortical layers of the visual cortex. Our analysis was centered on the neuronal populations of VISp, whilst also considering the neuronal populations in VISl and VISpm as points of reference against VISp. Due to a lack of data from Ntsr1-Cre GN220 mice for layer 6, our analysis did not include data from the posteromedial (VISal), rostrolateral (VISrl), and anteromedial (VISam) areas (Esfahany et al., 2018). Specifically, we employed the drifting grating stimulus session on the mouse visual cortex. We conducted experiments using mice with four cre-lines expressed in each of the layers. Cux2-Cre-ERT2 expressing on layers 2/3 and 4, Emx1-IRES-Cre and Slc17a7-IRES2-Cre expressing on layers 2/3, 4, and 5, and Ntsr1-Cre GN220 expressing on layer 6. As per de Vries et al. (2020), we utilized the representative depths for each layer: <250 μm (L2/3), 250–365 μm (L4), 375–500 μm (L5), and 550 μm (L6) from the surface. The dataset utilized a variety of visual stimuli, our focus was on the drifting grating stimulus, characterized by eight directions spaced evenly at 25° intervals, and five different temporal frequencies (1, 2, 4, 8, and 15 Hz). We also studied on the natural image stimulus that comprised 119 natural images. Each stimulus was displayed ~75 times. We selected experiments with neuron count greater than or equal to the specified neuron number. To isolate cellular objects and obtain the neural traces, a segmentation method that utilized the spatial and temporal information from the entire movie has been employed to get neuron traces of each experiment (Allen Institute MindScope Program, 2016). We analyzed each group of neurons separately and performed overall statistics on the population activity. By utilizing this dataset and applying our analysis methods, we were able to investigate the decoding characteristics and performance of neural populations in different layers of the mouse visual cortex.

#### 2.1.2. Stringer dataset

The Stringer dataset employed 2-photon imaging to capture neural activity, recording roughly 10,000 neurons concurrently through an 11-plane 3D scan at a 30 Hz time resolution. Multi-plane acquisition controlled by a resonance scanner was used, with planes spaced 30–35 μm apart in depth. This dataset presents findings from mice in which the GCaMP6s were expressed under the Emx1-IRES-Cre transgene's guidance, facilitating the expression of GCaMP6s from layers 2 to 5 in the mouse visual cortex. While various visual stimuli were integrated into the dataset, our analysis predominantly centered on the 32-class drifting grating stimuli and the natural image stimuli. For drift grating stimuli, the directions were evenly spaced at 11.25° intervals and had 32 directions with a spatial frequency of 0.05 cycles per degree and a temporal frequency of 2 Hz. For nature image stimulation, a set of 32 natural images was selected from the ImageNet (Russakovsky et al., 2015) database. Each stimulus was presented 70–120 times. All calcium signals were processed using Suite2p (Pachitariu et al., 2016). Compared to Allen's dataset, the *z*-axis data of the Stringer dataset was denser and contained data from more superficial layers, as Stringer's dataset began recording from 75 μm below the pial surface. Sequential acquisition of 10–12 planes was performed at a frequency of 3 or 2.5 Hz. For consistency, we only used data from 11 imaging depths and from 3 mouses: m31, m32, and m33 which underwent both of the two different stimulus experiments. Also, we used data from three mice that underwent both drift grating and nature image experiments. We excluded two groups of data for lack of data from 420 μm depth. We processed the data using the methods outlined in the Stringer paper (Stringer et al., 2019a), which included spontaneous components subtraction and normalization.

### 2.2. Decoding and quantification of decoding accuracy

For deciphering stimulus identity from neural reactions, multiple decoders were applied to the calcium traces. Aiming to evaluate the decoding accuracy across layers, the same decoders were consistently used within each dataset. For the Allen dataset, the techniques implemented included: (1) multi-class ECOC (error-correcting output codes) SVM (support vector machines), (2) k-NN (k-nearest neighbor), (3) decision tree (4) naive Bayesian (5) logistic regression. Regarding the Stringer dataset, we used SVM for decoder consistency and used a basic nearest-neighbor decoder according to Stringer's work (Stringer et al., 2019a). The nearest-neighbor decoder was based on pairwise correlations, assigning the stimulus identity as that with the highest correlation. This decoder suits datasets with a large number of neurons, as high-dimensional correlations can overwhelm the effects of high-amplitude responses that are unrelated to the stimulus exhibited by some of the neurons.

We employed almost identical data preprocessing methods for datasets. The amplitude of df/f traces for each trial was averaged to obtain a single value per stimulus, and then data were normalized across neurons for both datasets. For SVM, if not specially mentioned, we used 5-fold cross-validation with an 80/20 split to avoid selection biases. Decoding accuracy was defined as the fraction of correctly labeled stimuli. For each decoder, the selection of the neuron populations was repeated 15 times.

### 2.3. Quantification of neuron properties

To discern the properties influencing decoding accuracy among layers, multiple factors were quantified. The orientation selectivity index (OSI) measures a neuron's preference for its preferred orientation:


$$OSI=\frac{Rprefer-Rorthog}{Rprefer+Rorthog}$$

*Rprefer* and *Rorthog* are responses of each neuron to the preferred orientation and orthogonal orientation. OSI quantifies the selectivity of a neuron's response to visual stimuli with different orientations and is calculated as the difference between the neuron's responses to the preferred and non-preferred orientations, divided by the sum of the responses. A high OSI value indicates that the neuron is highly selective for a particular orientation and not widely tuned.

The direction selectivity index (DSI) measures a neuron's preference for its preferred direction of motion:


$$DSI=\frac{Rprefer-Ropposite}{Rprefer+Ropposite}$$

*Rprefer* and *Ropposite* are the responses of the neuron to the preferred direction of motion and the opposite direction of motion, respectively. DSI quantifies the selectivity of a neuron's response to visual stimuli with different directions of motion and is calculated as the difference between the neuron's responses to the preferred and opposite directions, divided by the sum of the responses. A high DSI value indicates that the neuron is highly selective for a particular direction of motion.

The reliability of neuronal responses measures the consistency and repeatability of the neuron's activity across repeated presentations of the same stimulus. It is commonly quantified using measures such as trial-to-trial correlation or reliability coefficient.


$$Reliability=\frac{2}{T^{2}-T}\overset{T}{\underset{i=1}{\sum }}\overset{T}{\underset{j=i+1}{\sum }}\rho \left(f_{i},f_{j}\right)$$

where *f*_*i*_ is the df/f response of the i-th trial of a cell's preferred condition. A higher reliability value indicates the neuron's more reliable and consistent response to the stimulus. The definitions of the above three properties were referenced from the neuronal property descriptions provided by the Allen Institute MindScope Program (2016).

The signal-to-noise ratio (SNR) is defined in this paper as the ratio of the amplitude responses of a single neuron to the same stimulus and the variance of the amplitude responses. The noise component represents the variation in response of a single neuron to the same stimulus. The SNR was calculated as:


$$SNR=\frac{\sum_{i=1}^{n}Ri^{2}-nVar(signal_{noise})}{nVar(signal_{noise})}$$

Where Ri is the amplitude response of a single neuron to the ith stimulus, *signal*_*noise*_ was defined as the unbiased estimation of the mean-variance in the responses of an individual neuron to different stimuli. Note that The SNR estimate is positive when a neuron responds to stimuli above its noise baseline.


$$Corr=\frac{\sum_{i=1}^{n}\left({\text{R1}}_{i}-\bar{\text{R1}}\right)({\text{R2}}_{i}-\bar{\text{R2}})}{\sqrt{{\sum_{i=1}^{n}\left({\text{R1}}_{i}-\bar{\text{R1}}\right)}^{2}}\sqrt{\sum_{i=1}^{n}{\left({\text{R2}}_{i}-\bar{\text{R2}}\right)}^{2}}}$$

where *R*1 and *R*2 are the trial-averaged responses of the two groups. The Pearson correlation coefficient between responses on the two repeats was used for the simple NN decoder. It measures the stability of population responses to the same stimulus.

### 2.4. Statistical analysis

Statistical evaluations in this research utilized two-sided Wilcoxon rank sum tests for distinct data and signed rank tests for paired observations. Significance levels were denoted as *p ≤ 0.05, **p ≤ 0.01, ***p ≤ 0.001, and ns, for *p* ≥ 0.05, indicating no significant difference. Importantly, the Wilcoxon rank sum tests did not presuppose a Gaussian distribution and were suitable for non-parametric data analysis. Outcomes with a *p*-value of ≤ 0.05 were deemed statistically significant. Unless specifically mentioned, all summarized data were presented as mean ± SEM. No outliers were discerned in the data points, ensuring their inclusion in the statistical assessments. In the graphical representation, relationships of no significance or relevance remained unlabeled.

## 3. Result

### 3.1. Differences in decoding accuracy across cortical layers of the visual cortex

**Figure 1:**
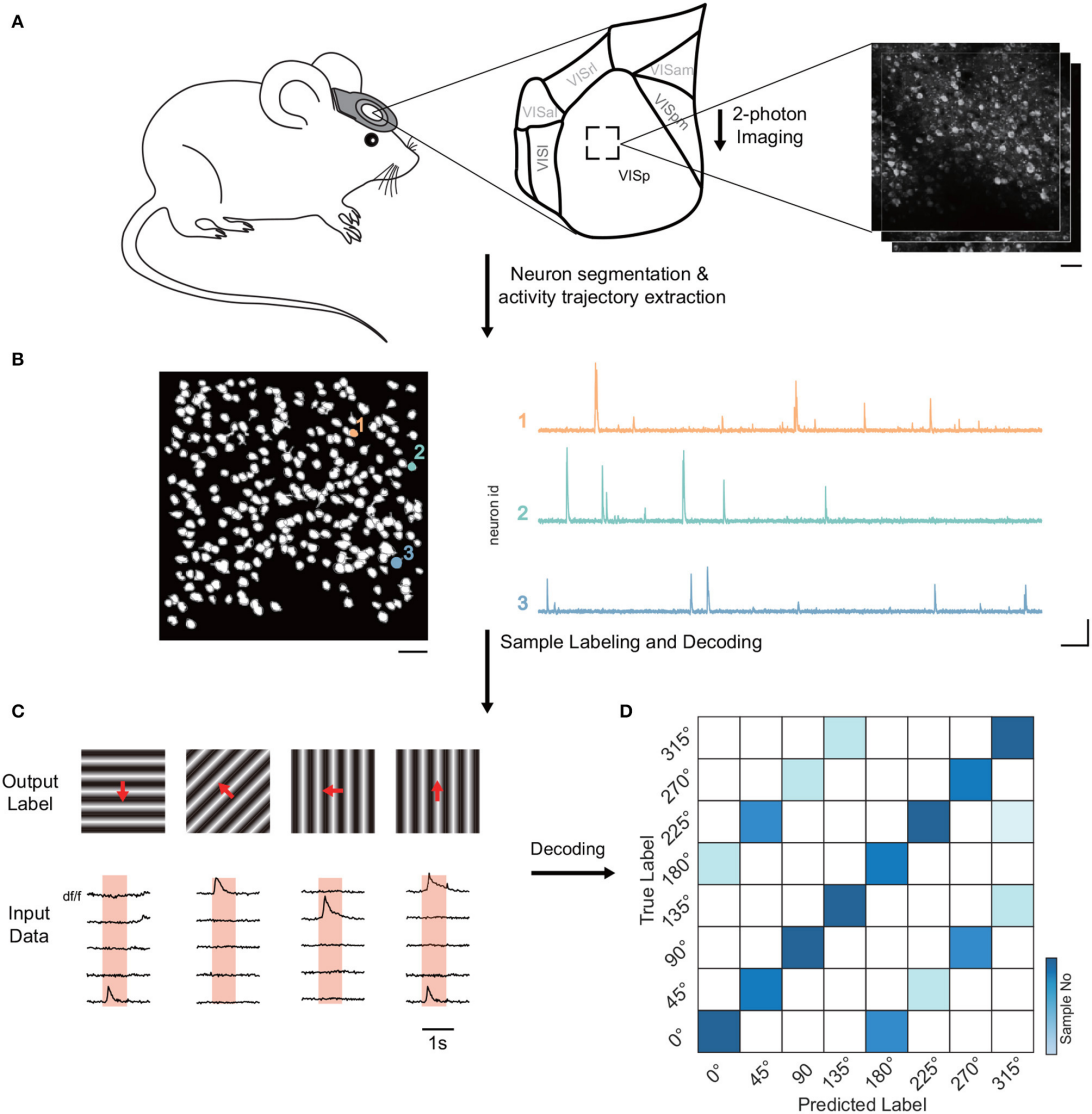
Diagram of pipeline for decoding visual stimuli in mice. **(A)** Experimental setup showcasing the recording of neuronal activity in the visual cortex of head-fixed mice via two-photon calcium imaging at varying imaging depths. The primary analysis was on VISp neuronal population. **(B)** Algorithms utilized for neuron activity extraction, detailing the segmentation of neurons and the extraction of activity traces. The scale bar denotes 40 μm (left), 10 s (right, horizontal), and 1 df/f (right, vertical). These respectively indicate the field of view, and temporal and amplitude scales. **(C)** Overview of input data (neuronal response) and output labels (stimulus identity) formulation. Input data is derived from the trial inner average per cell for each stimulus, while output labels showcase corresponding stimulus identities. Unless otherwise specified, 5-fold cross-validation with an 80/20 train-test split was applied. **(D)** Stimulus identity decoding from neural responses via multiple simple decoders like SVM, kNN, decision tree, logistic regression, naive Bayesian, and simple nearest neighbor correlation classifier. Data normalization was conducted across neurons, and 5-fold cross-validation was applied for data augmentation. The neuron selection process was repeated 15 times to address biases. The color gradient in the visualization signifies the number of samples.

Firstly, we examined Allen's dataset to discern the decoding accuracy among neural groups in varying layers of the mouse visual cortex (Figure 1A). The neural traces of the dataset were extracted based on the temporal and spatial components of the neurons (Figure 1B). Employing a range of decoders, we were able to predict the stimulus identity of each trial from the neural responses (Figures 1C, D). Decoding accuracy signified the ratio of accurately identified stimuli.

#### 3.1.1. Decoding neuron populations across cortical layers

**Figure 2:**
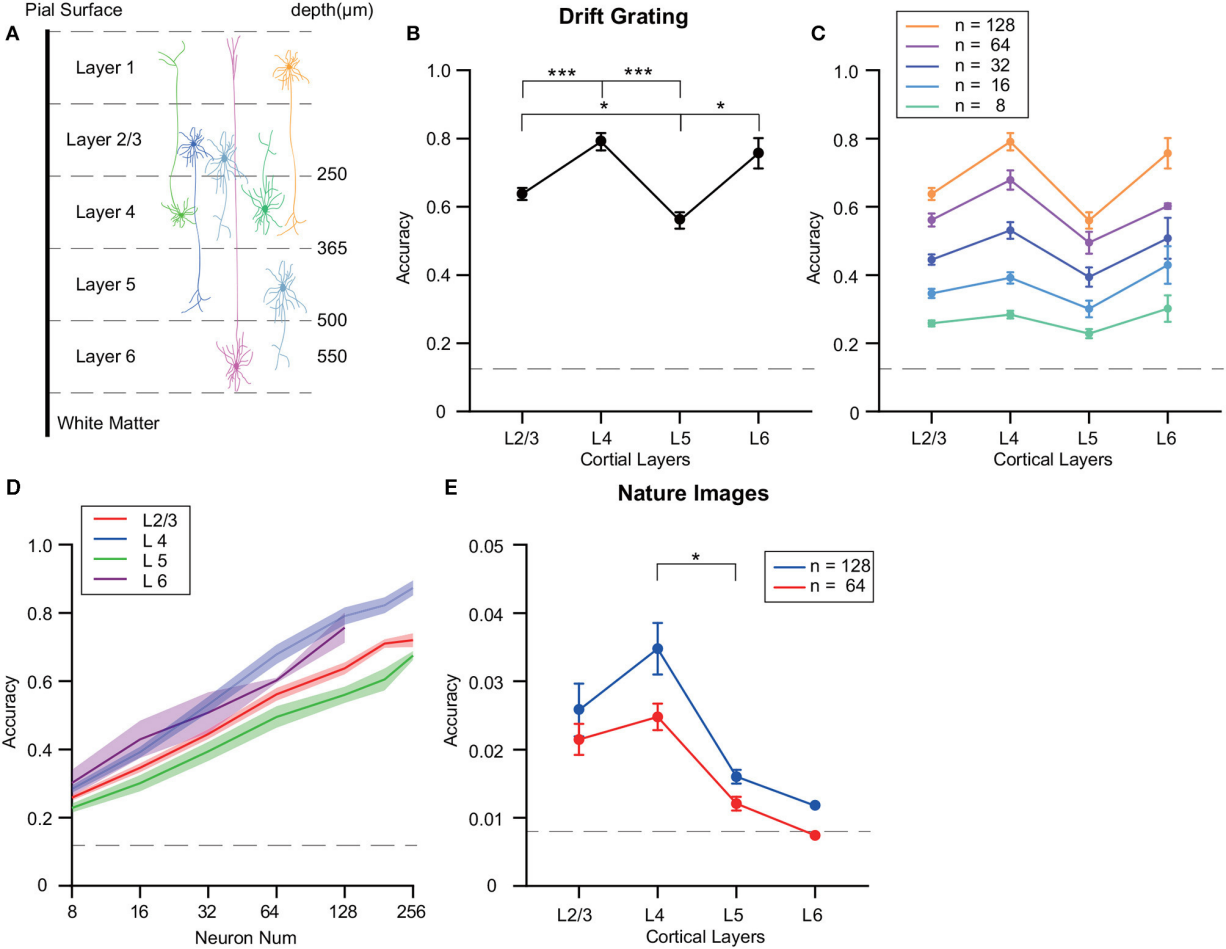
Differences in decoding accuracy of grating stimuli across different layers of the mouse visual cortex. **(A)** Illustrative scheme of the neuronal dendrites field across layers, spanning ~600 μm from the pial surface to the white matter. **(B)** Decoding accuracy analysis of VISp's various cortical layers using an SVM with the ECOC model. Focus was on neuronal populations within layers: L2/3, L4, L5, and L6. Notable differences in decoding accuracy are highlighted. **(C, D)** Inter-layer decoding variability investigation with diverse neuron population sizes using SVM. Neuron selection was iterated 10 times, with averaged results. L4 consistently maintained the highest decoding accuracy with neuron numbers more than 32, while L5 had the lowest accuracy. **(E)** Inter-layer decoding variability investigation with diverse neuron population sizes using SVM. Neuron selection was iterated 10 times, with averaged results. The decoding accuracy of L4 was significantly higher than L5, while the differences in decoding accuracy among the other layers were not statistically significant. *p ≤ 0.05, ***p ≤ 0.001.

The visual cortex exhibits a sequential order of sensory information transfer, along with variations in the distribution and projections of different neurons across its layers (Figure 2A). Such factors can lead to variability in decoding precision among the layers studied. To mitigate disparities due to neuron count in each test, we normalized the number of neurons utilized for precision assessment. Utilizing SVM for grating orientation decoding, we noticed fluctuating precision across neuron groups from disparate VISp layers, even with equivalent neuron counts (Figure 2B). Specifically, when decoding the drifting grating stimulus, we observed that L4 neurons exhibited significantly higher decoding accuracy compared to neurons in L2/3 and L5 (*p* ≤ 0.001 for L4, Wilcoxon rank sum test) and the decoding accuracy of the neuron population in layer 5 was significantly lower than that of L2/3 and L6 (*p* ≤ 0.05, Wilcoxon rank sum test).

Additionally, decoding performance was assessed by comparing sets of randomly selected neurons—ranging from 4 to 256—in each cortical layer experiment. Notably, L4 consistently maintained a high decoding accuracy for identical neuron counts, while L5 recorded the minimum accuracy (Figures 2C, D; Supplementary Figure 1A). Given the limited neuron count in the Allen dataset (Supplementary Table 1) and the observation that 128-neuron groups' decoding accuracy was non-saturating, we chose a uniformly sized group of 128 randomly chosen neurons for subsequent analyses.

Further, we executed decoding evaluations on natural image stimuli derived from the Allen dataset. For decoding 119 natural images, we compared decoding accuracy using 64 and 128 neurons across different layers. The results highlighted a significantly higher decoding accuracy of L4 compared to L5 (p ≤ 0.05) (Figure 2E).

### 3.2. Decoding performance of different decoders and cortical areas across layers

#### 3.2.1. Decoder type influences performance

**Figure 3:**
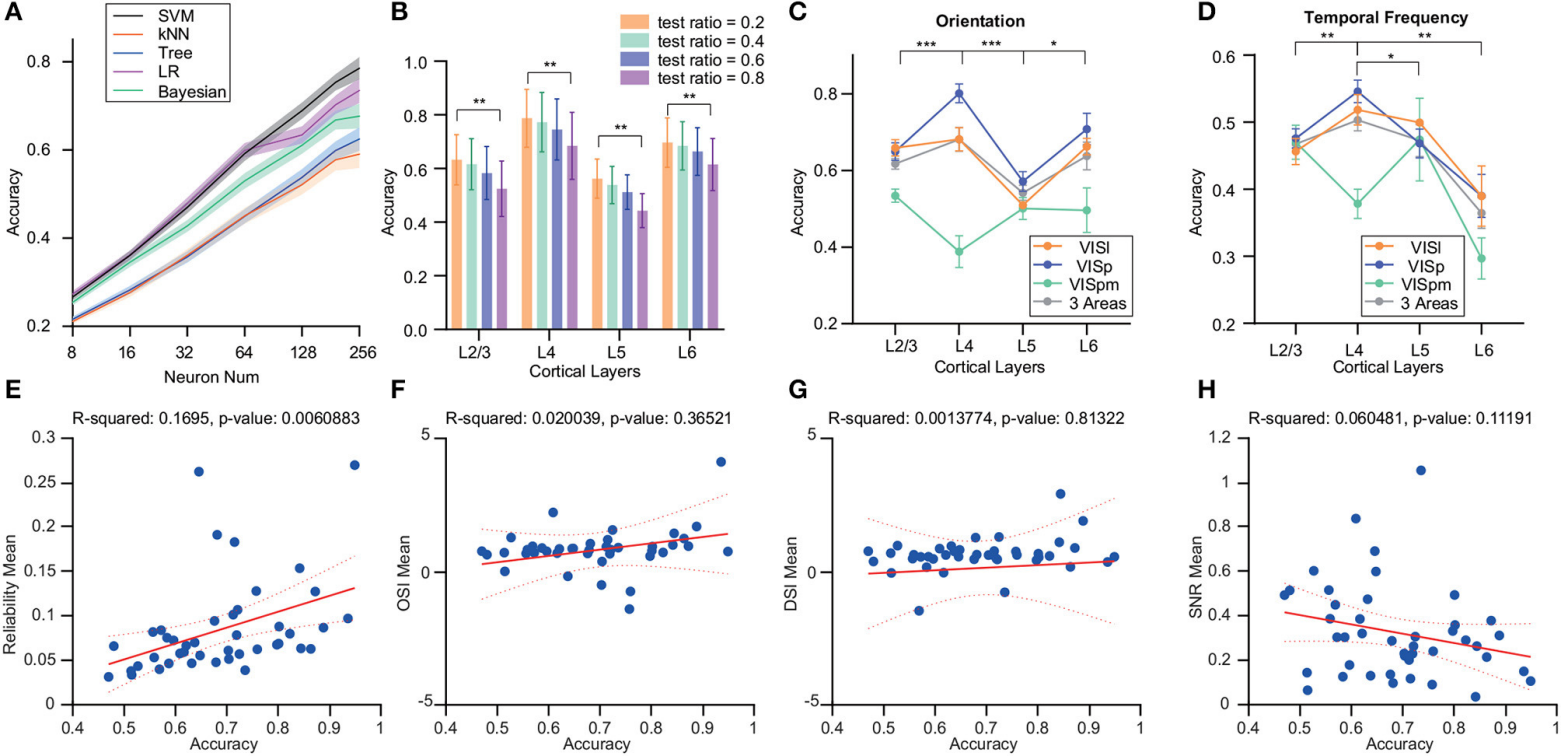
Analysis of the effects of different decoders and brain regions on decoding accuracy variations in the drift grating task across layers. **(A)** Deployment of multi-class ECOC model for SVM, kNN, binary decision tree, logistic regression, and naive Bayesian classifier for decoding. SVM showed superior decoding accuracy for stimulus identity. **(B)** Examination of the influence of varying train/test ratios on decoder performance. The test set ratio was finalized at 0.2 for subsequent evaluations. **(C)** Decoding accuracy differences for drift grating orientations decoding across different layers within various brain regions of the visual cortex. For VISp, accuracy was significantly higher for L4 than for L2/3 and L6 (*p* ≤ 0.05, *p* ≤ 0.001). In the VISl brain region, the decoding accuracy of L5 is significantly lower than the other cortical layers (*p* ≤ 0.05). The decoding accuracy of VISpm was lower than the other two brain regions, with L4 decoding significantly less accurately than L2/3 layers (*p* ≤ 0.05). **(D)** Differences in the accuracy of drift grating temporal-frequency decoding for different layers within different brain regions of the visual cortex. Compared with the decoding of grating drift orientations, the decoding accuracy of the L6 was lower. For VISp, accuracy was significantly higher for L4 than for other layers (*p* ≤ 0.05, *p* ≤ 0.01, *p* ≤ 0.001). The decoding accuracy of VISpm was lower than the other two brain regions, with L4 decoding significantly less accurately than L2/3 layers (*p* ≤ 0.05). All the significant markers were observed in VISp, indicating that the differences of interest between the other two brain regions will be addressed in the aforementioned descriptions. **(E–H)** The relationship between decoding accuracy and four factors: reliability, OSI, DSI, and SNR. Decoding accuracy showed a significant positive correlation with reliability. *p ≤ 0.05, **p ≤ 0.01, ***p ≤ 0.001 (Wilcoxon rank-sum test).

Firstly, we employed various decoders to analyze the decoder performance of neuron populations from VISp with different neuron numbers (Figure 3A). Notably, the logistic regression and SVM decoders outperformed others for up to 64 neurons. However, as neuron counts grew, SVM sustained its superior accuracy.

We also varied the proportions of training and test sets, maintaining a consistent total dataset size. This revealed a decline in decoding accuracy with decreasing training set proportions. However, this decrease did not affect the differences in decoding accuracy among cortical layers (Figure 3B).

#### 3.2.2. Difference in decoding performance varied across cortical areas

In addition to VISp, we assessed decoding accuracy in other areas like VISl and VISpm. Within VISp, L4 neurons displayed notably superior accuracy for grating orientation against other layers (*p* ≤ 0.001), while the decoding accuracy of L4 in VISl was not outstanding. In contrast, the decoding accuracy of VISpm's L4 was relatively lower compared to its L2/3 (*p* ≤ 0.05). Also, VISpm's decoding performance lagged behind the other two regions (Figure 3C).

Moreover, we observed that the decoding performance for temporal frequency was generally poorer compared to that for grating orientation even for just 5-class classification. Specifically, when decoding temporal frequency in VISp and VISl region, L6's decoding accuracy markedly trailed that of L4 (*p* ≤ 0.05). Within the VISpm area, L4's decoding accuracy for temporal frequency was noticeably lower compared to L2/3 (*p* ≤ 0.05) (Figure 3D).

#### 3.2.3. Influential factors on decoding accuracy

To explore the factors contributing to decoding disparities, we investigated the relationships between OSI/ DSI/ reliability/ SNR and decoding accuracy. We observed a significant positive correlation between decoding accuracy and reliability (Figure 3E). Yet, no discernible correlations were found between decoding accuracy and OSI, DSI, or SNR (Figures 3F–H). Although there is no significant linear relationship, the SNR of the neuronal population in Layer 4 is significantly higher than that of Layers 2/3 and 5 (Supplementary Figure 1B). Therefore, we delved deeper into the relationship between these neuronal characteristics and decoding accuracy.

### 3.3. Effect of stimuli and imaging depth variability on decoding accuracy

Due to the limitation of fixed imaging depth datasets, which do not allow comparison of SNR, OSI, and DSI across different depths within the same experiment, we utilized another 11-plane scanning imaging dataset (Stringer et al., 2019a). The dataset from Stringer permitted the examination of SNR, OSI, and DSI at multiple depths, providing additional insights into the neural properties in different cortical layers.

#### 3.3.1. Decoding performance across various imaging depths in stringer's dataset

**Figure 4:**
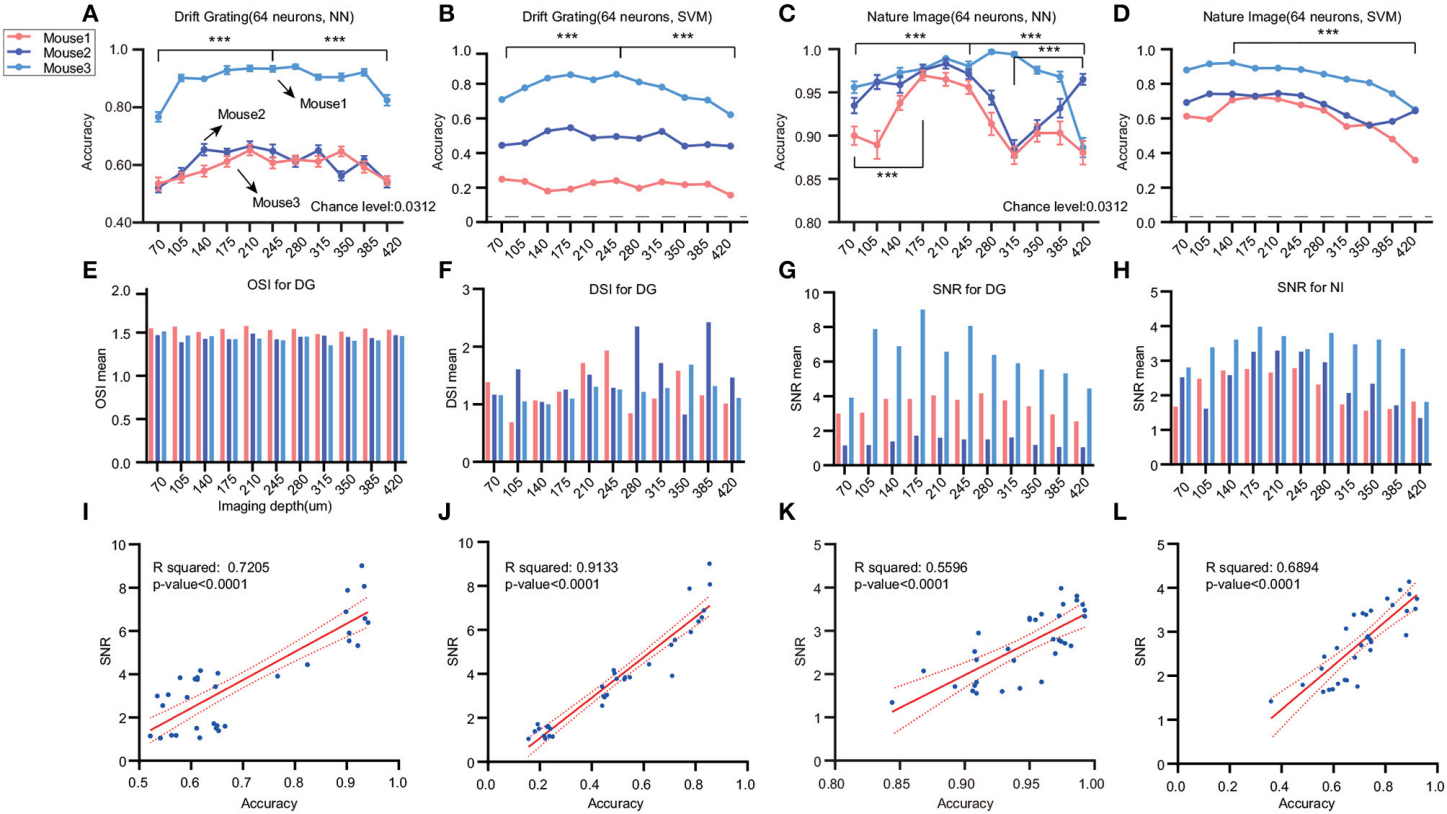
Effects of different stimuli and imaging depths on decoding accuracy. We analyzed the Stringer dataset to investigate the impact of diverse stimuli and imaging depths on decoding accuracy across different cortical layers. The dashed line represents the chance level. **(A, B)** Analysis of the drifting grating stimulus data with 11 imaging depths ranging from 70 to 420 μm (*n* = 3). We used NN and SVM to decode the 32 orientations. The colors correspond to different mice. **(C, D)** For the natural images stimulus data, we analyzed 11 imaging depths ranging from 70 to 420 μm (*n* = 3) (***p ≤ 0.001). The dashed line represents the chance level. **(E–H)** There are differences in DSI across layers, but the pattern is inconsistent. The SNR is higher in the middle layers and lower in the shallower or deeper locations. We filtered out OSIs > 5 to ensure the validity of the statistics. Also, the p-values of linear regression for DSI with all the decoding accuracy are larger than 0.05. **(I–L)** The relationship between SNR and decoding accuracy for different decoders and tasks are depicted in the figures, with corresponding *R*-square values and p-values displayed on the graphs. Except for OSI and SVM decoding in the DG task, all other results not mentioned on the graph have *p*-values > 0.05. The sample size is 33 for three mice and 11 cortical layers.

We conducted a further investigation using Stringer's dataset, which included data acquired at different imaging depths. Two types of stimuli were selected: drifting grating with 32 orientations and 32 natural images. We employed our previously validated optimal decoder, SVM, alongside the nearest neighbor (NN) decoder derived from Stringer's study. Upon analyzing data from the Drift grating stimulus, we observed that decoding accuracy exhibited a trend of being high in the middle layers and lower in the superficial and deep layers (Figures 4A, B). When analyzing the natural image data, we noted an increase in accuracy from the shallow to middle layers. However, in the case of mouse2, a decline in accuracy was noted from 210 μm down to 315 μm before showing a subsequent upward trend (Figures 4C, D). In summary, the decoding accuracy of drift grating stimuli showed an increasing trend with greater imaging depth from the pial surface down to 245 or 280 μm. Beyond this depth, the accuracy gradually decreased as the imaging depth became deeper. Considering that the Stringer dataset includes layers L2/3, L4, and L5, this finding aligns with the results obtained from the Allen dataset, indicating that within neuronal populations of the same size, L4 exhibits higher accuracy.

#### 3.3.2. Validation of influential factors on decoding accuracy

In the context of Allen's dataset, the SNR across various layers could be affected by both experimental conditions and inter-mouse variability, especially considering that data from diverse layers originate from separate experiments. This may seriously affect the observation of the intrinsic stimulus-tuning characteristics of SNR. 3D scanning offers an advantage for data analysis across different layers, as it enables the exploration of various layers under identical experimental conditions. To explore potential reasons for the observed decoding differences between drift grating (DG) and natural image (NI) stimuli, we examined individual neuron properties, including OSI, DSI, and SNR.

While OSI displayed limited variations across layers (Figure 4E), DSI revealed pronounced disparities between layers, albeit without a discernible distribution pattern (Figure 4F). Furthermore, no substantial linear correlation was identified between decoding accuracy across imaging depths and either OSI or DSI (Supplementary Figures 2A–F). Additionally, SNR values were elevated in the middle layers, compared to diminished values in both shallower and deeper layers, consistent across both stimuli (Figures 4G, H). Notably, there were significant positive linear relationships between the mean SNR and the decoding accuracy using both NN and SVM for DG orientations, as evidenced by the *R*-squared values (*R*-square 0.7205 for NN/DG, 0.9133 for SVM/DG, 0.5596 for NN/DG, 0.6894 for NN/DG) (Figures 4H–K). This finding suggests that SNR could be a contributing factor to the differences in decoding accuracy. It's pertinent to mention that disparities in SNR across experiments or layers cannot be ascribed to the recording technique, as Stringer et al. (2019b) have previously tested electrophysiology data using the same stimuli and obtained a similar distribution of SNR. Furthermore, our analysis of the noise levels in calcium signals across various neural layers did not mirror the trends observed in decoding accuracy or the stimulus-associated SNR (Supplementary Figures 2G–L).

## 4. Discussion

In our study, we contrasted the decoding accuracy of visual stimuli identity across neuronal populations from varying cortical layers within the mouse visual cortex. We assessed decoding disparities among neuron populations, ensuring consistent neuron numbers across layers. Additionally, we evaluated the performance variations among different simple decoders employed for this task. We employed 5-fold cross-validation to bolster our data, averaging results through the iterative selection of a consistent neuron number to balance discrepancies arising from neuron selection. We chose simple structured decoders because they provide faster computation speeds, better interpretability, increased robustness, enhanced generalization capabilities, and reduced risk of overfitting compared to complex models, making them highly suitable for decoding neuronal population activities in neuroscience research. SVM outperformed other decoders in accuracy as neuron numbers increased. This is determined by the inherent characteristics of SVM. Firstly, due to the high feature dimensionality of neuron populations (e.g., larger than 100), SVM can find a hyperplane to separate different classes. Also, considering the presence of noise in neuron responses, the regularization technique of SVM effectively mitigates overfitting and minimizes the impact of noisy data on the classifier. Additionally, SVM based on the ECOC model is designed for handling multi-class classification tasks by decomposing the problem into multiple binary classifiers with unique error-correcting codes, providing benefits such as effective handling of complex datasets, reduced misclassification errors, improved generalization, and interpretable classification process, making it well-suited for our tasks.

Furthermore, we examined the decoding differences among neuronal populations in different cortical layers across various brain regions within the visual cortex. Our results indicated that the decoding accuracy of neuron population in L4 VISp for grating stimuli was higher than that of other layers with no significant differences in L4 and L6, and lower accuracy was observed in L5. The higher decoding accuracy of the neuronal population in L4 may be attributed to its direct reception of first-hand information from the thalamus following sensory information processing (Sun et al., 2016). Moreover, decoder logic indicates that the neuron populations are more conducive for stimulus identity decoding if responding to a wide range of stimuli or not responding to any stimuli. Yildirim et al. (2019) utilized three-photon microscopy to investigate the single-neuron response properties in different cortical layers of the visual cortex in awake mice. Yildirim et al. reported that neurons in L5 exhibit a broader orientation tuning compared to neurons in other cortical layers. This finding was also supported by multi-cortical electrophysiological recordings (Niell and Stryker, 2008) and L5-specific two-photon studies (Lur et al., 2016). The broader tuning of L5 neuronal populations could explain the lower decoding accuracy observed in this layer. Regarding the L6 neuronal population, despite a potential deterioration in the signal-to-noise ratio with increasing imaging depth, it still exhibited good decoding performance. This can be attributed to the presence of a large number of cortico-thalamic projection neurons in L6, which are known to possess a high degree of orientation selectivity (Vélez-Fort et al., 2014). This property of L6 also supports that the decrease in the number of neurons has a relatively smaller impact on the decoding accuracy of L6 compared to other cortical layers. While our study yielded significant conclusions supported by multiple experiments, further attempts with larger datasets and more microscopic data results could strengthen our findings.

For other brain regions, Esfahany et al. (2018) study reported higher OSI and DSI values for individual neurons in VISl. In our validation, we observed comparable decoding accuracy between VISl and VISp. This similarity could be attributed to the previously mentioned relatively low correlation between population decoding performance and OSI or DSI. Alternatively, it could be influenced by the less redundant organization of excitatory neurons in VISp (Latham and Nirenberg, 2005). On the other hand, the neural population of VISpm exhibited lower decoding performance compared to VISp, which could be attributed to the narrower and broader stimulus preferences in VISp as supported by Marshel et al. (2011) study. Additionally, it is important to note that the inter-layer differences we demonstrated only suggest that L4 neuronal populations of the same neuron number retain relatively more information. It does not directly prove that all neurons within that layer retain more information, as we did not obtain information from every single neuron in each layer. Therefore, it is still possible that neurons in other layers, such as L5 and L6, may exhibit sparse distribution and potentially retain significant information.

Notably, while two-photon imaging offers the advantage of high spatial resolution 3D imagery via tissue scanning, it demands a trade-off in temporal resolution and necessitates intricate apparatus. Thus, we chiefly concentrated on data from the Allen Brain Observatory, given its use of high temporal resolution imaging and a larger group of experiments. However, as Stringer's dataset was acquired using 3D scanning, we can control the impact of experimental conditions and individual differences among mice on SNR. In our analysis of Stringer's dataset, we found that decoding with neurons in shallow layers (75μm under the pial surface) may not be as accurate as with neurons in deeper imaging depths (175–280 μm), despite previous demonstrations that neurons in shallow layers decode better (Esfahany et al., 2018). Additionally, we identified the signal-to-noise ratio as a contributing factor to the variation in decoding accuracy, alongside the OSI and DSI. The stimulus-related SNR and reliability serve as a measure of response quality and stability, which holds significant importance for any decoder, particularly the SVM and NN decoders utilized in our study. Also, NN performs particularly well when the size of the neuronal population is large, making it highly suitable for Stringer's data.

Although there were some differences in the data analysis compared to some of the previous studies, this research provided new evidence for the hierarchical structure of information processing in the mouse visual cortex. The results confirmed the inter-layer differences in visual information processing. These findings contribute to our understanding of the functional organization of the visual cortex and have implications for the development of the visual cortex. Further studies are needed to explore the precise mechanisms underlying the observed inter-layer differences in population decoding and to investigate their potential relevance to visual perception and behavior.

## Data availability statement

The original contributions presented in the study are included in the article/Supplementary material, further inquiries can be directed to the corresponding author.

## Author contributions

YW and GX initiated and supervised the project. YW and CK conceptualized the research and methodology. CK conducted data analysis and drafted the manuscript, with assistance from YW and GX. The research was discussed by all authors, who contributed to the article and approved the final version for submission.
