# Supplementary material for: Neuron populations across layer 2-6 in the mouse visual cortex exhibit different coding abilities in the awake mice
Source: Front Cell Neurosci. 2023 Sep 25;17:1238777. doi: 10.3389/fncel.2023.1238777 (PMC10560757; doi:10.3389/fncel.2023.1238777)
Supplement: Supplementary file 1 [file Data_Sheet_1.pdf]

Supplementary Material

1 SUPPLEMENTARY TABLES AND FIGURES

1.1 Table

Table S1. The detail information of two dataset including GCaMP variants, cre line of mice, trial number and mice number of each cre line, neuron number of each trail, target cell, and presense in which cortical layers. Note that in Allen Brain Observatory dataset, L2/3 data were collected less than 250 μm from the surface, L4 data were collected between 250 and 365 μm, L5 data were collected between 375 and 500 μm were assigned to layer 5, and L6 data were collected at 550 μm as stated in de Vires's paper.

Dataset (GCaMP/Area)	Cre line	Trial num /Mice num	Neuron num (mean±std)	Target cell (Excitory or not)	Layers
Allen (GCaMP6f) DG(VISp)	Cux2-CreERT2	16/13	222.4 ± 67.3	Excitory	L2/3, 4
	Emx1-IRES-Cre	10/7	219.9 ± 62.8	Excitory	L2/3, 4, 5
	Ntsr1-Cre-GN220	6/6	67.7 ± 56.3	Excitory	L6
	Slc17a7-IRES2-Cre	20/17	225.0 ± 104.0	Excitory	L2/3, 4, 5
Allen (GCaMP6f) DG(VISl)	Cux2-CreERT2	13/11	170.5 ± 73.3	Excitory	L2/3, 4
	Emx1-IRES-Cre	8/6	180.8 ± 9.7	Excitory	L2/3, 4, 5
	Ntsr1-Cre-GN220	3/3	94.0 ± 64.9	Excitory	L6
	Slc17a7-IRES2-Cre	12/10	158.1 ± 97.7	Excitory	L2/3, 4, 5
Allen (GCaMP6f) DG(VISpm)	Cux2-CreERT2	14/12	124.9 ± 53.3	Excitory	L2/3, 4
	Emx1-IRES-Cre	3/2	125.33 ± 47.0	Excitory	L2/3, 4, 5
	Ntsr1-Cre-GN220	4/4	73.3 ± 39.8	Excitory	L6
	Slc17a7-IRES2-Cre	10/9	86.1 ± 24.8	Excitory	L2/3, 4, 5
Allen (GCaMP6f) NI(VISp)	Emx1-IRES-Cre	10/7	189.4 ± 52.6	Excitory	L2/3, 4, 5
	Ntsr1-Cre-GN220	5/5	68.8 ± 41.6	Excitory	L6
	Slc17a7-IRES2-Cre	18/16	209.8 ± 78.5	Excitory	L2/3, 4, 5
Allen (GCaMP6f) NI(VISl)	Emx1-IRES-Cre	8/6	169.4 ± 86.3	Excitory	L2/3, 4, 5
	Ntsr1-Cre-GN220	3/3	90.6 ± 49.5	Excitory	L6
	Slc17a7-IRES2-Cre	12/10	153.0 ± 100.4	Excitory	L2/3, 4, 5
Allen (GCaMP6f) NI(VISpm)	Emx1-IRES-Cre	4/2	120.7 ± 60.6	Excitory	L2/3, 4, 5
	Ntsr1-Cre-GN220	4/4	66.5 ± 33.6	Excitory	L6
	Slc17a7-IRES2-Cre	11/10	78.6 ± 27.4	Excitory	L2/3, 4, 5
Stringer (GCaMP6s)	Emx1-IRES-Cre	6/3	10537 ± 1672.9	Excitory	L2/3, 4, 5

1.2 Figures

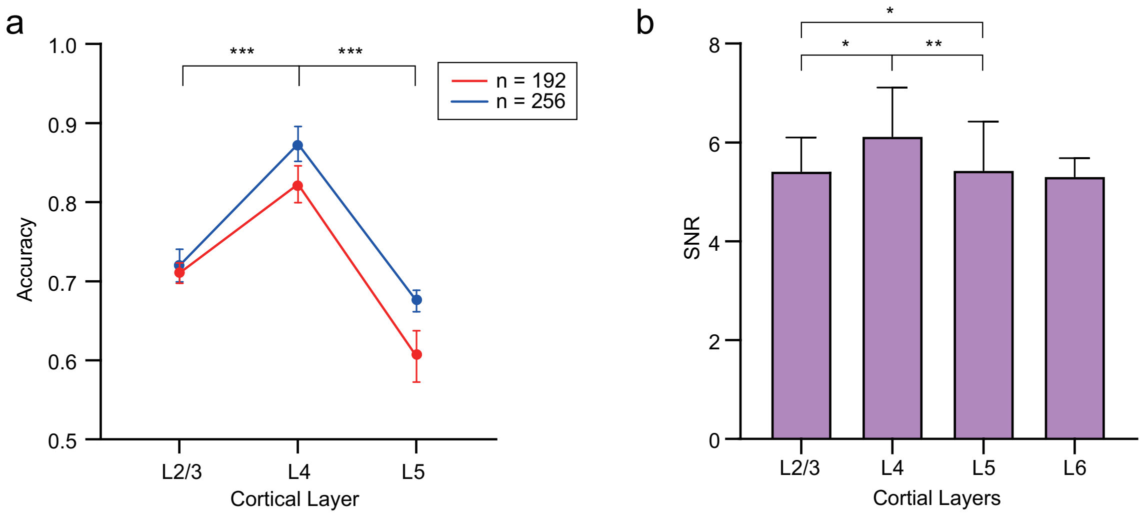

Supplementary Figure S1. **(a)** Decoding accuracy difference for drift grating orientations using the Allen dataset, with neuron populations selected to include more than 192 neurons and 256 neurons. The sixth group did not have experiments with more than 192 neurons. Decoding accuracy of L4 was significantly higher than L2/3 and L5 (***$p \leq 0.001$). **(b)** Distribution of SNR in the Allen dataset(*$p \leq 0.05$, **$p \leq 0.01$).

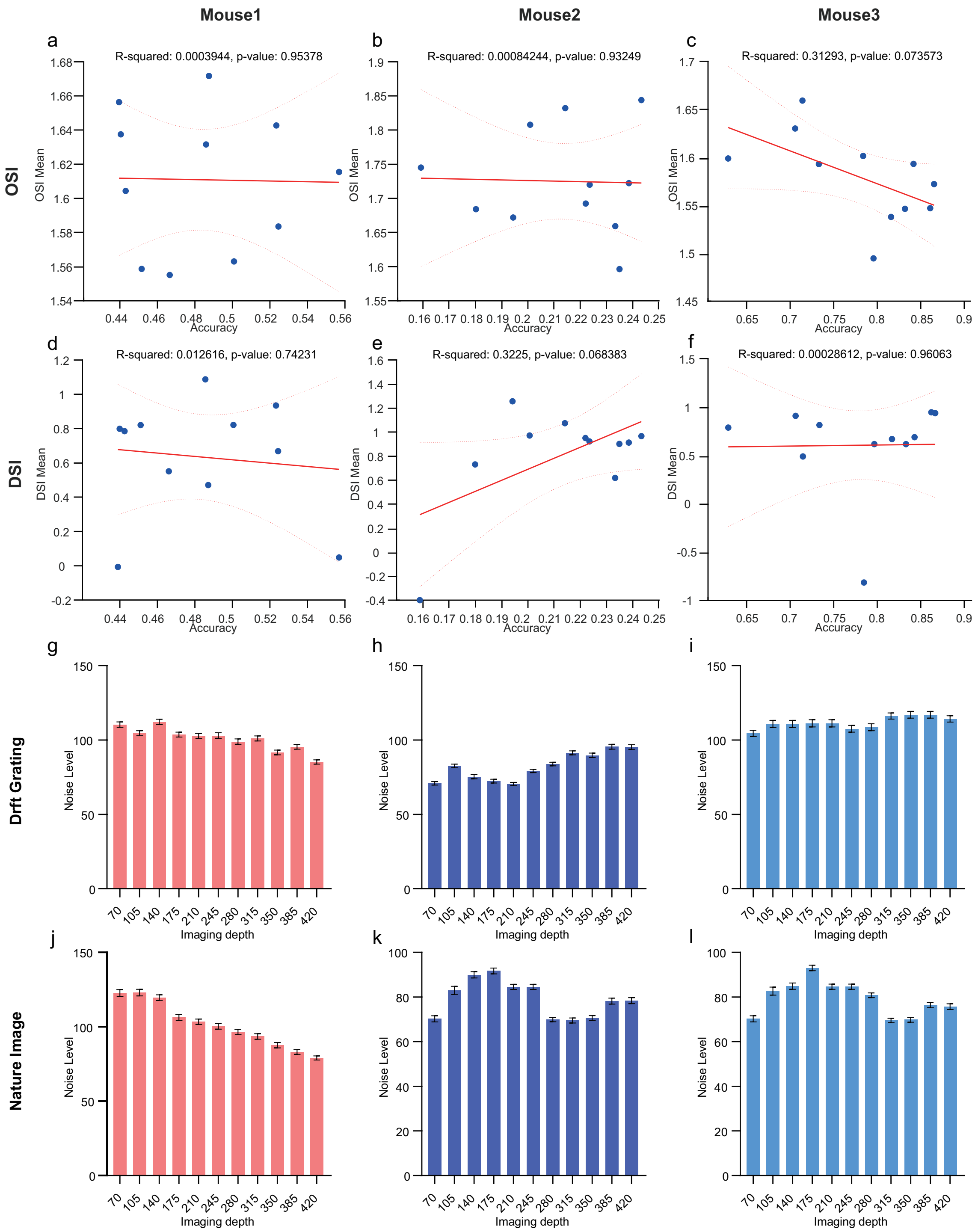

Supplementary Figure S2. **(a-f)** The SVM decoding accuracy of orientation for Drift Grating stimuli in the Stringer dataset does not show a significant linear relationship with OSI. The dataset includes 11 cortical layers (70um, 105um, 140um, 175um, 210um, 245um, 280um, 315um, 350um, 385um, 420um), and the linear relationships between SVM decoding accuracy and OSI/DSI are indicated by R square values, with significance denoted by p-values. **(g-l)** The noise level changes with imaging depth. The noise level was calculated according to Suite2p. In one mouse, the noise level shows a decreasing trend with increasing imaging depth, but there is no overall trend of being higher in the middle layers and lower in both superficial and deep layers.
